# Reference Gene Selection for Quantitative Real-Time PCR of Mycelia from* Lentinula edodes* under High-Temperature Stress

**DOI:** 10.1155/2018/1670328

**Published:** 2018-06-11

**Authors:** Xu Zhao, Huanling Yang, Mingjie Chen, Xiaoxia Song, Changxia Yu, Yan Zhao, Yuejin Wu

**Affiliations:** ^1^Institute of Technical Biology & Agriculture Engineering, Hefei Institutes of Physical Science, Chinese Academy of Sciences, Hefei 230031, China; ^2^University of Science and Technology of China, Hefei 230026, China; ^3^Institute of Edible Fungi, Shanghai Academy of Agricultural Sciences, Shanghai 201403, China; ^4^College of Life Sciences, Nanjing Agricultural University, Nanjing 210095, China

## Abstract

Housekeeping genes are important for measuring the transcription expression of functional genes; 10 traditional reference genes,* TUB, TUA, GADPH, EF1, 18S, GTP, ACT, UBI, UBC,* and* H2A*, were tested for their adequacy in* Lentinula edodes* (*L. edodes*). Using specific primers, mRNA levels of these candidate housekeeping genes were evaluated in mycelia of* L. edodes*, which were treated with high-temperature stress at 37°C for 0, 4, 8, 12, 18, and 24 hours. After treatment, expression stability of candidate genes was evaluated using three statistical software programs: geNorm, NormFinder, and BestKeeper. According to geNorm,* TUB* had the lowest M values in* L. edodes* strains 18 and 18N44. Using NormFinder, the best candidate reference gene in strain 18 was* TUB* (0.030), and the best candidate reference gene in strain 18N44 was* UBI* (0.047). In BestKeeper analysis, the standard deviation (SD) values of* UBC*,* TUA*,* H2A*,* EF1*,* ACT*,* 18S*, and* GTP* in strain 18 and those of* GADPH* and* GTP* in strain 18N44 were greater than 1; thus, these genes were disqualified as reference genes. Taken together, only* UBI* and* TUB* were found to be desirable reference genes by BestKeeper software. Based on the results of three software analyses,* TUB* was the most stable gene under all conditions and was verified as an appropriate reference gene for quantitative real-time polymerase chain reaction in* L. edodes* mycelia under high-temperature stress.

## 1. Introduction

Quantitative real-time polymerase chain reaction (qRT-PCR), which is characterized by high sensitivity, strong specificity, a repetitive dynamic quantitative range and high throughput, is one of the most commonly used techniques for gene expression analysis. The accuracy of qRT-PCR results is largely dependent on the selected reference genes [1–6], the validity of which is a prerequisite for the correct application of qRT-PCR to analyze changes in target gene expression [7–9]. To obtain more accurate and reliable results, reference genes are required for standardized measurement of the expression levels of target genes [10]. In recent years, considerable research has shown that the choice of reference genes should be associated with the experimental conditions of the investigator. The selection of reference genes may change depending on experimental conditions [1, 2, 11–13]. In general, a basic component of the cytoskeleton or a relatively stable expressed gene involved in basic metabolic processes is selected as the reference gene. Nonetheless, research has shown that the expression levels of such stably expressed genes may not be stable in various cell types, at different developmental stages or under different experimental conditions [14–17]. Therefore, the evaluation and selection of reference genes under different experimental conditions are of great significance for obtaining reliable and precise qRT-PCR results [18, 19].

*L. edodes* is a Basidiomycete which belongs to the class Agaricomycetes, order Agaricales, and the family Tricholomataceae [20].* L. edodes*, also known as shiitake, is a medical and edible fungus [21, 22]. This mushroom fructifies under moderate and low temperatures, with an optimal temperature for spore germination between 22 and 26°C, for mycelial growth between 24 and 27°C, for primordium differentiation between 10 and 12°C, and for fruit body development between 8 and 20°C [22]. If the temperature is below its optimal value for mycelial growth,* L. edodes* will grow more slowly, and mycelia will be thicker. Poor growth may occur both below 0°C and above 32°C, whereas growth cessation and terminal death occur at 35°C and 38°C, respectively. If the temperature is above the optimum, the fruit body grows faster, with a thin cap and a long stipe that result in a loose texture, an easy-to-open umbrella, and poor quality. In this study,* L. edodes* mycelia were subjected to high-temperature stress for various time periods. Ten genes were selected as candidate reference genes, and three types of software (geNorm, NormFinder, and BestKeeper) were used to evaluate these candidates as reference genes for* L. edodes* under high-temperature stress. The results of this study lay the foundation for future research regarding molecular mechanisms of* L. edodes* in response to high-temperature stress.

## 2. Materials and Methods

### 2.1. Material and Processing

*L. edodes* strains 18 and 18N44 were obtained from the Institute of Edible Fungi, Shanghai Academy of Agricultural Sciences.

Mycelia of* L. edodes* were incubated and then treated with high-temperature stress at 37°C for 4, 8, 12, 18, and 24 hours; individuals not exposed to high-temperature stress were used as the control group. Biology duplications are three times. The mycelia were collected under sterile conditions, flash-frozen in liquid nitrogen, and stored at -80°C.

### 2.2. Extraction and Purification of Mycelia Total RNA

Total RNA of mycelia was extracted with a Redzol reagent kit from Beijing SBS Genetech Co., Ltd., according to the instruction manual. RNA integrity was assessed by 1% agarose gel electrophoresis at 120 V for 10 minutes, and RNA purity was measured using a NanoDrop 2000 C (Thermo Fisher Scientific, Hudson, New Hampshire, USA).

### 2.3. Selection of Reference Genes and Primer Design

Candidate reference genes were selected according to previous literature [23–27]:* TUB*,* TUA*,* GADPH*,* EF1*,* 18S*,* GTP*,* ACT*,* UBI*,* UBC*, and* H2A*. These genes were annotated with the NCBI genome database of* L. edodes* strain W1-26 (NCBI LDAT00000000, SRS875031, and SRS1090734) or the genome database of* L. edodes* (http://legdb.chenlianfu.com/) [28] and subsequently used in primer design.

The primers were designed using the NCBI online server tool with an amplification product length ranging from 70 to 300 bp. All primers were synthesized by the Shanghai Generay Biotech Co., Ltd., and the primer sequences are presented in Table 1.

### 2.4. Evaluation of Amplification Efficiency of Candidate Reference Genes

Using* L. edodes* mycelia that were not treated with high-temperature stress (hereafter referred to as CK) for comparison, the primer pairs were amplified to detect amplicon production using cDNA as the template. The successfully amplified bands products were retrieved and ligated to a T-vector, and genes were sequenced by Sangon Biotech.

The amplification efficiency of the reference genes was calculated referring to the method of Li Peng [27]. The cDNA template of CK was diluted, and the cycle threshold (Ct value) was determined based on qRT-PCR. Calibration curves were prepared based on logarithmic fitting with the cDNA dilution and Ct value as the abscissa and ordinate, respectively. The amplification efficiency of each primer pair was calculated according to relevant data, with three repeats for each specimen.

### 2.5. Reverse Transcription (RT) of cDNA and Quantitative Real-Time (qRT) PCR

This experiment was performed on ice using Prime Script RT Reagent Kit with gDNA Eraser (Takara, Shiga, Japan) according to the manufacturer's instructions. cDNA acquired by reverse transcription was stored at -20°C. qRT-PCR was performed using the SYBR®* Premix Ex Taq*™ II (Takara, Shiga, Japan) Kit and a StepOnePlus Real-Time PCR instrument. The reaction system included 10 *μ*L SYBR®* Premix Ex Taq*™ II (Tli RNaseH Plus) (2x), 0.4 *μ*L ROX dye, 2 *μ*L template cDNA, 0.4 *μ*L each primer, and 6.8 *μ*L RNase-free water. The PCR amplifications were performed using a 7500 Real-Time PCR System (Applied Biosystems, Foster City, CA, USA) using the following: predegeneration at 95°C for 30 s; PCR at 95°C for 5 s, 60°C for 15 s, and 72°C for 15 s for 40 cycles; melting curve at 95°C for 15 min, 60°C for 30 s, and 95°C for 15 min.

qRT-PCR was performed on cDNA from high-temperature-sensitive and high-temperature-tolerant stains 18 and 18N44 under heat stress treatment and Ct values were recorded. The data were later imported into geNorm software, and the minimum value of Ct under different treatments was set to 1. Hence, the relative expression of other reference genes should be as follows: 2^-ΔΔCt^ (ΔCt = Ct-1, Ct value of each reference gene) [29]. The threshold M was set to less than 1.5, and NormFinder and BestKeeper were used for additional analyses.

### 2.6. Validation of Reference Genes

In order to confirm the validity of the selected reference genes for data normalization, the expression profiles of 10 candidate reference genes were synthetically analyzed and sorted. Two most stable and one least stable candidate reference genes were selected. In the meanwhile, expression levels of hydrophobin gene (*hyd1*) and heat shock protein 100 gene (*hsp100*) under high-temperature stress were verified by qRT-PCR analysis.

The qRT-PCR amplification conditions were the same as those described above. The relative expression levels of the two genes were calculated by 2^-ΔΔCt^ method [30, 31]. The analysis of gene expression by qRT-PCR was performed using three technical replicates for each biological replicate.

## 3. Results

### 3.1. Total RNA Quality

In this study, total RNA of* L. edodes* mycelia was extracted using the Redzol reagent method and a NanoDrop 2000 for detection of concentration and quality. The OD260/OD280 of the extracted RNA ranged from 1.8 to 2.2, and 1.0% agarose gel electrophoresis performed on the RNA indicated good quality. The quality of RNA is a crucial factor for fluorescence quantitative analysis. As shown in Supplementary Figure S1, the 28S, 18S, and 5S subunit bands were clear and the fragments were intact, with no obvious degradation; therefore, the material was used in follow-up experiments.

### 3.2. Amplification Efficiency and Specificity of Candidate Reference Gene Primers

PCR was performed to confirm the amplification capacity of all candidate reference gene primers. The results indicated a single band for all 10 pairs of primers; the amplified products were in line with the size of the expected fragment, and no primer dimers appeared. The PCR products were purified, and the sequencing results were consistent with reference sequences, which also found no similarity with other genes by Nucleotide-BLAST in the whole-genome shotgun contigs (WGS) database for strain W1-26. Verified by gradient PCR, all 10 pairs of primers showed excellent amplification at an annealing temperature approximately 60°C.

### 3.3. Calculation of Amplification Efficiency of Candidate Reference Genes

The cDNA from CK samples were diluted into five concentration gradients, with each sample repeated three times. qRT-PCR was performed for all 10 candidate reference genes. There were no impurity peaks in the dissociation curve, i.e., only the main peak was observed (Supplementary Figure S2). The Ct values of the candidate reference genes were evaluated using the dilution concentration of cDNA as the abscissa and the Ct value as the ordinate on the logarithmic fitting calibration curve. Thus, the regression coefficients of reference genes were calculated in accordance with the formula *E* = 10^-1/slope^-1, and amplification efficiency can be determined based on the slope of the curve. The results showed that the amplification efficiency of each candidate reference gene fell between 95% and 110%. The value of R^2^ was greater than 0.99, indicating a single dissolution curve. qRT-PCR was therefore performed using the ΔΔCT method (Table 2).

### 3.4. geNorm Analysis Results

**Figure 1:**
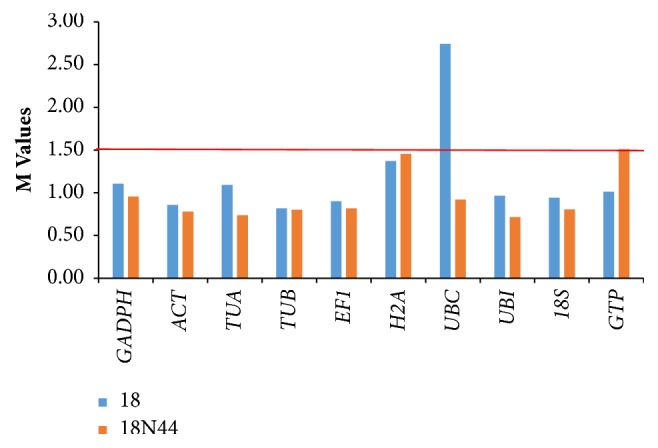
Analysis of 10 reference genes using geNorm. Low M values indicate high expressional stability. In iterative steps, genes with the lowest stability (i.e., the highest M value) are removed. When the threshold value of M is smaller than 1.5, the candidate reference parameters are applicable. The two less stable genes (*UBC* and* GTP*) are eliminated.

For geNorm analysis, we followed the methods of Estchmann and others [32]. The minimum value of Ct of a specific reference gene was set to 1, and the relative expression of other reference genes was expressed using the 2^-ΔCt^ formula. By calculating the Ct values of the 10 reference genes of samples, M can be determined using geNorm. When the threshold value of M is smaller than 1.5, the candidate reference parameters are applicable. A stable expression map of candidate reference genes from two strains of* L. edodes* under high-temperature stress over different time periods is illustrated in Figure 1. The M value of the 10 genes in 18 and 18N44, including* TUB*,* TUA*,* GADPH*,* EF1*,* 18S*,* GTP*,* ACT*,* UBI*,* UBC*, and* H2A*, was determined using geNorm software. Based on the results,* UBC* at 2.742 in strain 18 and* GTP* at 1.508 in strain 18N44 exceeded the threshold, whereas the other reference genes were comparatively stable.

**Figure 2:**
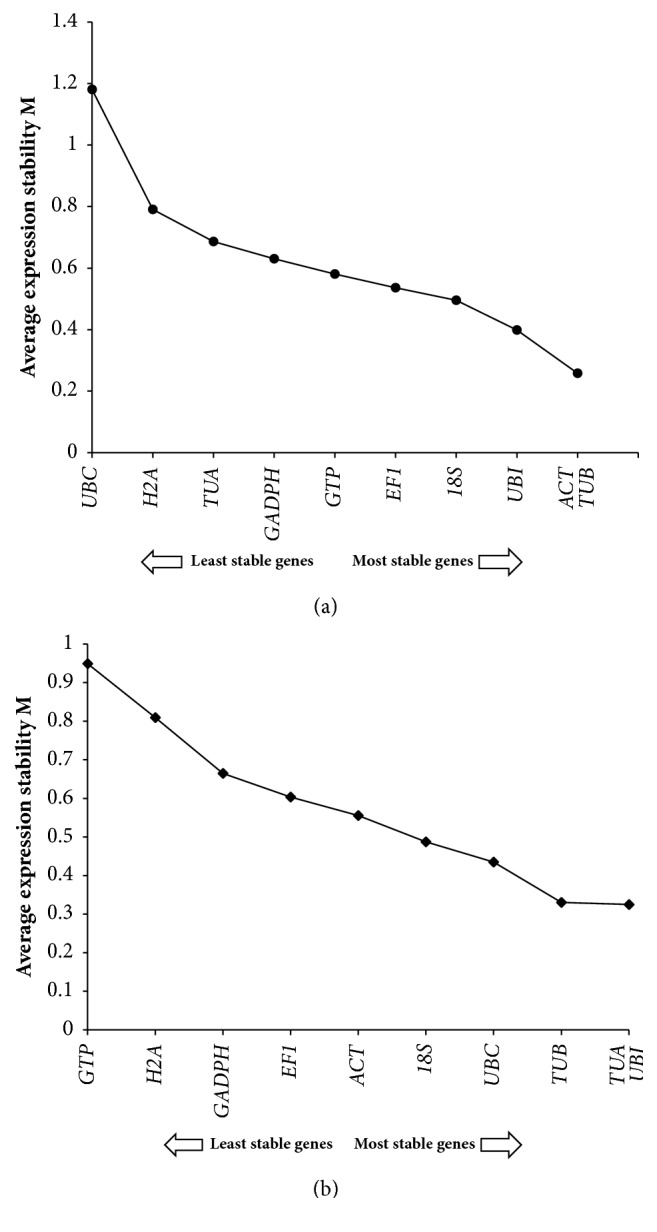
Gene expression stability of 10 reference genes ranked according to geNorm analysis. Gene expression studies for identification of most stable reference genes under high-temperature stress using geNorm software based on average expression stability value (M). The direction of arrow indicates the most and least stable reference genes in graphs. (a) strain 18; (b) strain 18N44.

The stability of these genes was evaluated using material from strains 18 and 18N44. With regard to strain 18,* ACT* and* TUB* showed high stability, followed by* UBI* and* 18S*; in contrast,* UBC* exhibited low stability (Figure 2(a)). Differences in the stability of the reference genes in strain 18N44 were also examined, with* TUB*,* TUA*, and* UBI* showing the most stable expression (Figure 2(b)).* TUB* displayed the lowest M values in both strains of* L. edodes*.

### 3.5. NormFinder Analysis Results

geNorm software can not significantly differentiate reference genes that share similar expression patterns. However, NormFinder software can effectively avoid unfavorable analysis results in this situation. NormFinder software conducts expression stability analysis on candidate reference genes mainly based on the results of variance analysis, and the software then lists genes in accordance with their stability [33]. The analysis of the expression stability of the 10 candidate reference genes in* L. edodes* at 37°C for different durations is presented in Table 3; lower values indicate more stable expression. The results showed that the best candidate reference gene in strain 18 was* TUB* (0.030); that in strain 18N44 was* UBI* (0.047).

### 3.6. BestKeeper Analysis Results

**Figure 3:**
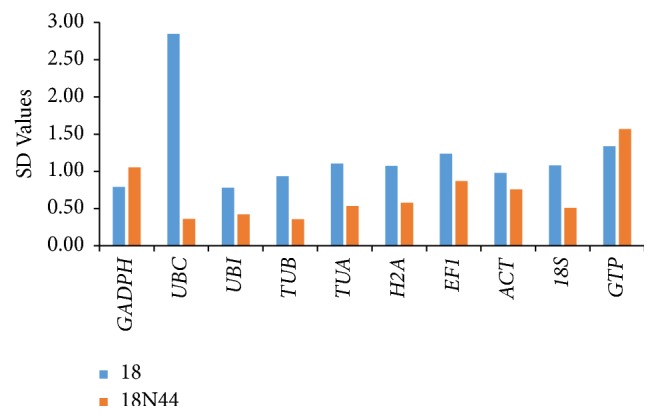
Analysis of 10 reference genes SD values using BestKeeper. Reference genes with an SD below 1 are considered stably expressed, and a smaller SD indicates a more stable reference gene. The results showed that* UBI* and* TUB* are stable genes (SD < 1).

The standard deviation (SD) and coefficient of variation (CV) as well as a pair of correlation coefficients (Poisson correlation coefficient) were calculated by BestKeeper via paired correlation analysis. Reference genes with an SD below 1 are considered stably expressed, and a smaller SD indicates a more stable reference gene [34]. BestKeeper analysis (Figure 3) revealed SD values greater than 1 for* UBC*,* TUA*,* H2A*,* EF1*,* ACT*,* 18S*, and* GTP* in strain 18 and* GADPH* and* GTP* in strain 18N44, disqualifying them as reference genes. Hence, only* UBI* and* TUB* were found to be desirable reference genes.

### 3.7. Determination of the Optimal Number of Reference Genes for Normalization

**Figure 4:**
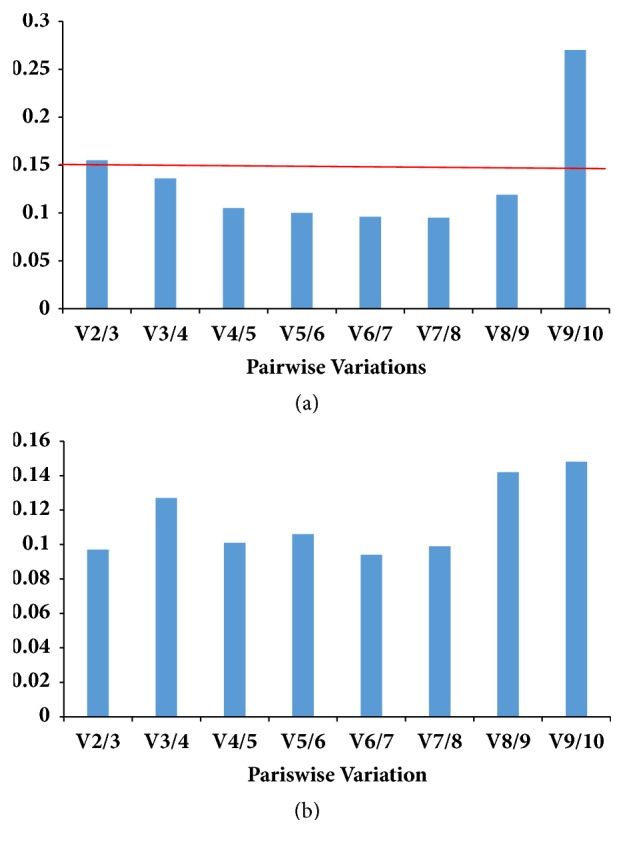
Pairwise variation (Vn/n+1) values calculated by geNorm. The cut-off value to determine the optimal number of reference genes for qRT-PCR normalization is 0.150. Vn/n+1 < 0.15 indicates that the inclusion of an additional reference gene is not required. (a) strain 18; (b) strain 18N44.

Some reports have suggested that the use of more reference genes may lead to more stable results [13, 35]. To determine the optimal number of reference genes in each experimental condition, pairwise variation (V) was calculated using geNorm by applying a cut-off value of 0.150 [13]. The pairing variable value Vn/n+1 can be analyzed using geNorm software to determine the optimal reference gene under certain conditions. In this study, Vn/n+1 was V3/4<0.150 (0.136, Figure 4(a)) in strain 18, indicating that three reference genes are sufficient as a standardized indicator. The variable value satisfied the condition of V2/3<0.150 (0.097, Figure 4(b)) in strain 18N44, suggesting that two reference genes should be used for normalization.

According to NormFinder software analysis, the most stable reference gene in strain 18N44 is* UBI*, followed by* TUA* and* TUB*; in strain 18, the most stable reference gene is* TUB*, followed by* EF1* and* UBI*. Analysis using BestKeeper (Table 4) software indicated that smaller SD and CV values were correlated with better outcomes. If the SD value exceeded 1, it was insufficient as a reference gene; hence, the eligible candidate reference genes were* GADPH*,* TUB*, and* TUA*. Based on the above analyses,* TUB* was chosen as the most suitable reference gene.

### 3.8. Validation of Reference Genes

**Figure 5:**
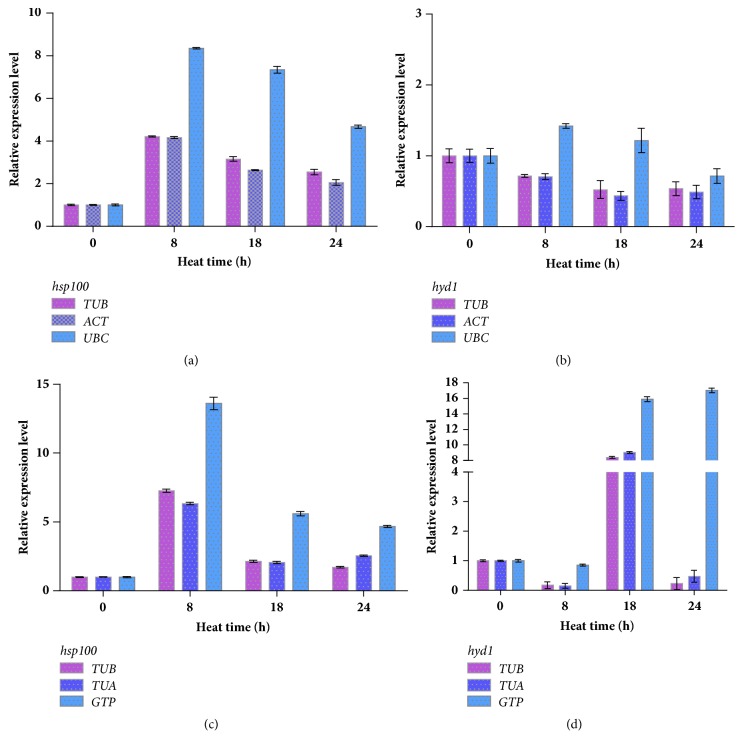
Validation of the selected reference genes. Relative expression of* hsp100* and* hyd1* in mycelia of* L. edodes* strains 18 (a; b) and 18N44 (c; d). In the strain 18,* TUB* and* ACT* were the most stable candidate reference genes, and* UBC* was the least stable candidate reference gene. In the strain 18N44,* TUB* and* TUA* were the most stable candidate reference genes, and* GTP* was the least stable candidate reference gene. Data are shown as mean ± standard deviation (n = 3).

To validate the selection of candidate reference genes, the relative expression of two key genes* hsp100* and* hyd1* in response to high-temperature stress were monitored. The results revealed that the two genes were differentially or specifically expressed (Figure 5).

The above comprehensive analysis indicates that, in the strain 18,* TUB* and* ACT* were the most stable candidate reference genes, and* UBC* was the least stable candidate reference gene. From Figures 5(a) and 5(b), relative expression levels of* hsp100* and* hyd1* obtained by normalizing against* TUB* or* ACT* indicated similar trends. However, normalization against* UBC* led to the opposite conclusion, exhibiting that expression of the two genes was relatively higher under high-temperature stress at 37°C for 8, 18, and 24 hours.

In the strain 18N44,* TUB* and* TUA* were the most stable candidate reference genes, and* GTP* was the least stable candidate reference gene. From Figures 5(c) and 5(d), relative expression levels of* hsp100* and* hyd1* obtained by normalizing against* TUB* or* TUA* indicated similar trends. However, normalization against* GTP* led to the opposite conclusion, exhibiting that the two genes were relatively higher under high-temperature stress at 37°C for 8, 18, and 24 hours.

## 4. Discussion

In qRT-PCR analysis, certain differences in the expression stability of the same reference gene can be observed among various species. Therefore, prior to expression analysis of a target gene, it is necessary to evaluate the expression stability of reference genes under certain experimental conditions [11, 33, 36]. The ideal reference genes should have unaltered expression levels during the entire experimental process and with proper expression intensity [3]. However, as there is no single reference gene that is appropriate for all experimental conditions [37], it is necessary to examine the expression stability of reference genes at different stages [38–40] or under different biotic and abiotic stresses to assess their use for normalization [41–43].

A stable reference gene is a prerequisite for improving the reliability of qRT-PCR results. The selection of reference genes was once mainly based on the functions of housekeeping genes. For instance,* Actin* and* TUB* are the basic components of the cytoskeleton, whereas* GAPDH*,* EF1a*,* UBQ*, and other proteins are involved in fundamental metabolism, which suggests that these genes are stably expressed in all cells and physiological states [44, 45]. Later studies showed that expression of housekeeping genes is not necessarily consistent in different species under the same stress condition [46]. For example, in this study,* TUB* was stably expressed after high-temperature treatment of mushrooms, and* Fb15* and* UBQ5* were found to be the best reference genes for gene expression studies in rice endosperm under high temperature [47]. Regarding the selection of optimal reference genes of plants under high-temperature stress, the research conducted by Yan Haidong and others [48] revealed that* eIF4A*,* TBP-1*, and* E2* could be used as reference genes for perennial ryegrass under high-temperature stress.* 26S rRNA* [49] and* EF1a* [50] have the most stable expression in wheat and maize, respectively, after high-temperature treatment. In addition,* SmEF1a* and* SmUCP* represent the most stably expressed reference genes of the eggplant heat-sensitive strain 05-1 and heat-resistant strain 05-4, respectively, at various time intervals during high-temperature stress [47]. To our knowledge, the selection and validation of reference genes under high-temperature stress in* L. edodes* have not been evaluated. In this study, expression levels as well as the stability of 10 candidate reference genes were measured in* L. edodes* using 3 different algorithms. The three algorithm yielded quiet different results with respect to the ranking of the 10 candidate reference genes, indicating the importance of using more than one software to achieve the best results. Due to the different algorithms of the three analysis software, there was a slight difference in the ranking of candidate reference genes, but with the comprehensive analysis of* TUB*, it is still the best choice as a reference gene under high-temperature stress. In order to verify the result, two genes* hsp100* and* hyd1*, which are related to fungal resistance to high-temperature stress, were selected. The two best candidate reference genes and the worst candidate reference gene were used, respectively; the quantitative results of* hsp100* and* hyd1* were compared and verified. It was found that, when the two most stable candidate reference genes (*TUB* and* ACT* in strain 18;* TUB* and* TUA* in strain 18N44) were used, they have a similar expression trend, but while the least stable candidate reference gene (*UBC* in strain 18 and* GTP* in strain 18N44) was used, its expression of* hsp100* or* hyd1* had significantly higher than that of the two most stable internal reference gene, which would further prove the accuracy of* TUB* as a good reference gene for* L. edodes* under high-temperature stress. These results further implied that the use of an unstable reference gene could lead to the misinterpretation of gene expression results [51].

Therefore, choosing the best reference gene largely depends on the relevant factors such as species and tissue type, as well as experimental conditions. Different tissues and organs, growth and developmental stages, and biotic or abiotic stress as well as hormonal status will often, if not always, exert a certain influence on the specific expression of reference genes.

## 5. Conclusion

This study investigated the expression stability of reference genes of different* L. edodes* strains that have undergone high-temperature stress over different time periods. The 10 genes (*TUB, TUA, GADPH, EF1, 18S, GTP, ACT, UBI, UBC,* and* H2A*) examined in this work are commonly studied reference genes. Specific amplification of the designed primers was confirmed via several methods including melt curve analysis with single peaked dissociation curves, the results of gel electrophoresis, with all products being single bands with no primer dimers, and the sequencing results which were consistent with reference sequences. In the qRT-PCR experiments, all 10 primer pairs had acceptable amplification efficiency, indicating that the qRT-PCR results are reliable. Stability analysis of candidate reference genes was mainly based on the analysis using geNorm software [52–54]. Combined with NormFinder and BestKeeper, the data for selecting an optimal reference gene was further supported. The results showed that* TUB* stands out as a reference gene that was stably expressed in the two strains 18 and 18N44 from* L. edodes*.

Overall, this research may provide a reference for analyzing differences in gene expression in* L. edodes* under stress conditions, as well as for a prerequisite study of the molecular mechanism of* L. edodes* in response to high-temperature stress. More importantly, being able to accurately measure changes in gene expression is an essential step for the selection of new* L. edodes* strains more tolerant to high-temperature stress.

## Figures and Tables

**Table 1:** Primer sequences of candidate reference genes.

Symbol	Gene name	Annotation	Primer sequence (forward/reverse)	Size (bp)
*18S*	18S ribosomal RNA	LE01Gene00881.t1	GCAACATCAAAACCTGGCGT GACATTCGACAACGCCCAAC	164
*EF1*	Elongation factor	LE01Gene03252.t2	CGGTGTTGTCAAGGGAAAGA GGTGACGATCATTCCAGCTT	178
*ACT*	Actin	LE01Gene01050.t1	GTGTTACCCATACCGTTCCC ATCGGTCAAATCACGACCAG	89
*GAPDH*	Glyceraldehyde-3-phosphate dehydrogenase	LE01Gene07114.t1	GTTCCTACCCTCGACGTTTC GATCGAAGAGTGGTTGTCCC	180
*GTP*	GTP-binding protein	LE01Gene00681.t1	ACCATCGTTCACACTCCCAC GGGAAGCTCGAACTTGGGAA	267
*H2A*	Histones H2A	LE01Gene03840.t1	TTTGTATCCGTGTCGTGTCC TGGGACTATCGCACATTTCC	108
*TUA*	Alpha-tubulin	LE01Gene01434.t1	CGACGAGATCAGGAATGGTC TTCCGATGGTGTAGTGACCT	110
*TUB*	Beta-tubulin	LE01Gene08622.t3	GACATTTGCTTCCGAACCCT CGGACATAACAAGGGACACA	79
*UBI*	Ubiquitin-protein ligase E3	LE01Gene00676.t1	CGTCGGGTTCTACGAGAAAG GTTCCGTGCTTTGTTCCTTG	133
*UBC*	Ubiquitin-conjugating	LE01Gene00040.t1	CTTGGAAATGAGTGGTCCCC TTGCGGGTCTCCATTAAACC	187

**Table 2:** Slopes of qRT-PCR curves for reference genes.

Gene	Equation of standard curves	Slope	Regression coefficient	Amplification efficiency
*GAPDH*	y = -3.131x + 31.876	-3.131	R^2^ = 0.999	108.654%
*18S*	y = -3.226x + 23.333	-3.226	R^2^ = 0.993	104.142%
*ACT*	y = -3.277x + 21.576	-3.277	R^2^ = 0.996	101.927%
*UBI*	y = -3.302x + 23.212	-3.302	R^2^ = 0.990	100.86%
*GTP*	y = -3.262x + 29.435	-3.262	R^2^ = 0.999	102.559%
*H2A*	y = -3.185x + 23.602	-3.185	R^2^ = 0.996	106.054%
*TUA*	y = -3.287x + 17.442	-3.287	R^2^ =0.997	101.483%
*UBC*	y = -3.198x + 21.166	-3.198	R^2^ =0.996	105.454%
*EF1*	y = -3.244x + 17.020	-3.244	R^2^ =0.998	103.367%
*TUB*	y = -3.274x + 28.293	-3.274	R^2^ =1	102.048%

**Table 3:** Expression stability of reference genes analyzed by the NormFinder algorithm.

Rank	Gene	18 Stability	18N44 Stability
1	*GADPH*	0.270	0.167
2	*ACT*	0.076	0.115
3	*TUA*	0.139	0.102
4	*TUB*	0.030	0.130
5	*EF1*	0.057	0.200
6	*H2A*	0.108	0.473
7	*UBC*	0.186	0.120
8	*UBI*	0.065	0.047
9	*18S*	0.114	0.171
10	*GTP*	0.082	0.212
Best Gene		*TUB*	*UBI*

**Table 4:** CP data for candidate reference genes.

	*GADPH* HKG1	*UBC* HKG2	*UBI* HKG3	*TUB* HKG4	*TUA* HKG5	*H2A* HKG6	*EF1* HKG7	*ACT* HKG8	*18S* HKG9	*GTP* HKG10
n	12	12	12	12	12	12	12	12	12	12
GeoMean[CP]	17.93	21.50	19.74	18.80	15.96	23.02	18.12	16.59	24.05	23.48
arMean[CP]	17.97	21.91	19.85	18.83	15.99	23.18	18.18	16.72	24.08	23.56
min[CP]	16.19	17.66	17.20	17.25	14.70	19.77	15.41	13.40	22.23	20.07
max[CP]	20.09	30.95	23.20	21.08	17.89	27.97	20.47	20.30	26.55	27.20
Std dex[±CP]	0.95	4.00	2.04	0.90	0.91	2.52	1.14	1.77	1.00	1.61
CV[%CP]	5.26	18.24	10.27	4.77	5.67	10.88	6.28	10.61	4.16	6.83
min[x-fold]	-3.35	-14.32	-5.80	-2.93	-2.93	-9.53	-6.57	-9.13	-3.54	-10.62
max[x-fold]	4.46	702.42	11.04	4.83	3.83	30.83	5.10	13.12	5.63	13.19
Std dex [±x-fold]	1.93	15.96	4.11	1.86	1.88	5.75	2.21	3.42	2.00	3.05

## Data Availability

The dataset supporting the conclusions of this study is available and the authors have agreed to share the dataset. The readers can contact them by email to obtain the raw data in their manuscript.

## Abbreviations

*L. edodes*: * Lentinula edodes*
qRT-PCR: Quantitative real-time polymerase chain reaction
*18S*: 18S ribosomal RNA
*EF1*: Elongation factor
*ACT*: Actin
*GAPDH*: Glyceraldehyde-3-phosphate dehydrogenase
*GTP*: GTP-binding protein
*H2A*: Histones H2A
*TUA*: Alpha-tubulin
*TUB*: Beta-tubulin
*UBI*: Ubiquitin-protein ligase E3
*UBC*: Ubiquitin-conjugating.

## Supplementary Materials

Supplementary Materials Supplementary Figure S1: electrophoresis gel of RNA samples from 18 (A) and 18N44 (B). The 28S, 18S, and 5S subunit bands were clear and the fragments were intact, with no obvious degradation, and quality was good; therefore, the material was used in follow-up experiments. Supplementary Figure S2: the qRT-PCR amplicon specificity of candidate reference genes. Melting curves generated by qRT-PCR. For each subgraph, temperature is displayed in the x axis; the derivative reporter signal is displayed in the y axis. Dissociation curves with single peaks generated for all amplicons.
