# Supplementary material for: Reference Gene Selection for Quantitative Real-Time PCR of Mycelia from Lentinula edodes under High-Temperature Stress
Source: Biomed Res Int. 2018 Jun 11;2018:1670328. doi: 10.1155/2018/1670328 (PMC6016149; doi:10.1155/2018/1670328)
Supplement: Supplementary Materials — Supplementary Figure S1: electrophoresis gel of RNA samples from 18 (A) and 18N44 (B). The 28S, 18S, and 5S subunit bands were clear and the fragments were intact, with no obvious degradation, and quality was good; therefore, the material was used in follow-up experiments. Supplementary Figure S2: the qRT-PCR amplicon specificity of candidate reference genes. Melting curves generated by qRT-PCR. For each subgraph, temperature is displayed in the x axis; the derivative reporter signal is displayed in the y axis. Dissociation curves with single peaks generated for all amplicons. [file 1670328.f1.docx]

**Supplementary Information：**


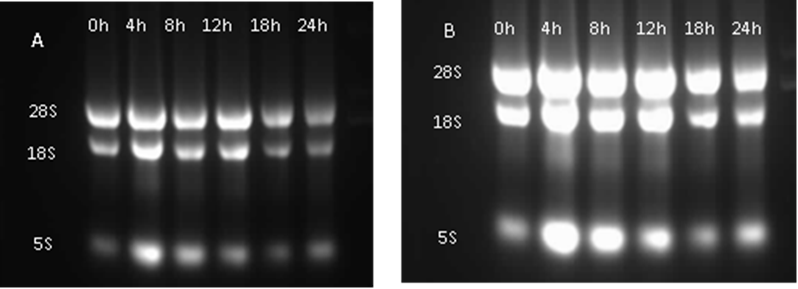


**Supplementary Figure S1：** Electrophoresis gel of RNA samples from 18 (A) and 18N44 (B). The 28S, 18S and 5S subunit bands were clear and the fragments intact, with no obvious degradation, and quality was good; therefore, the material was used in follow-up experiments.


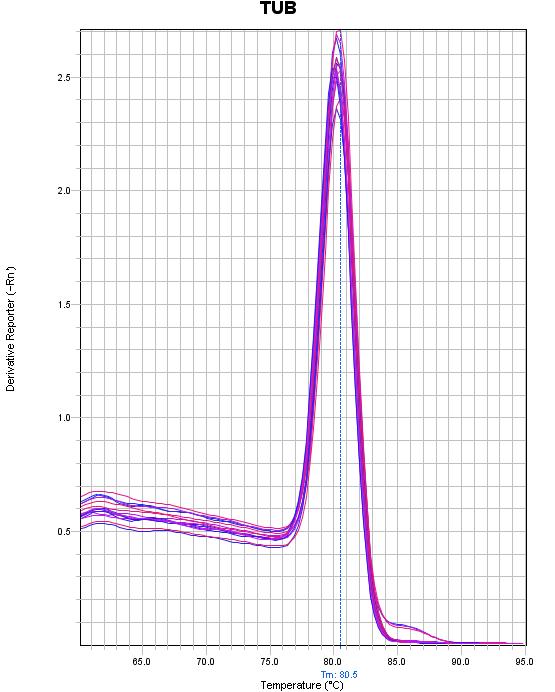

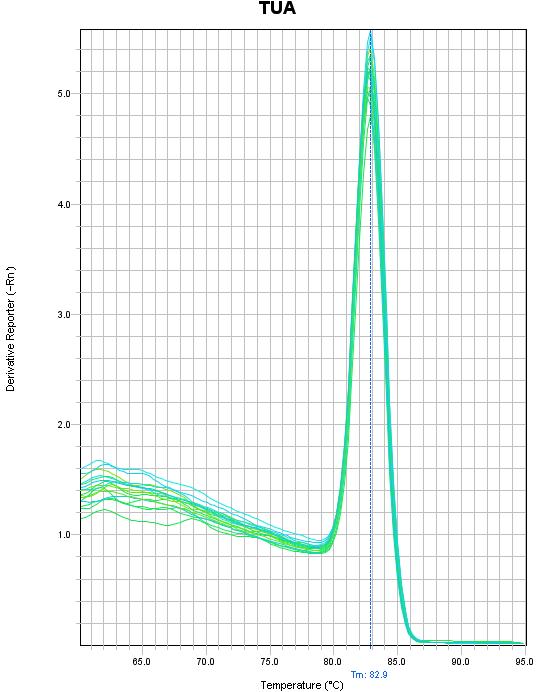

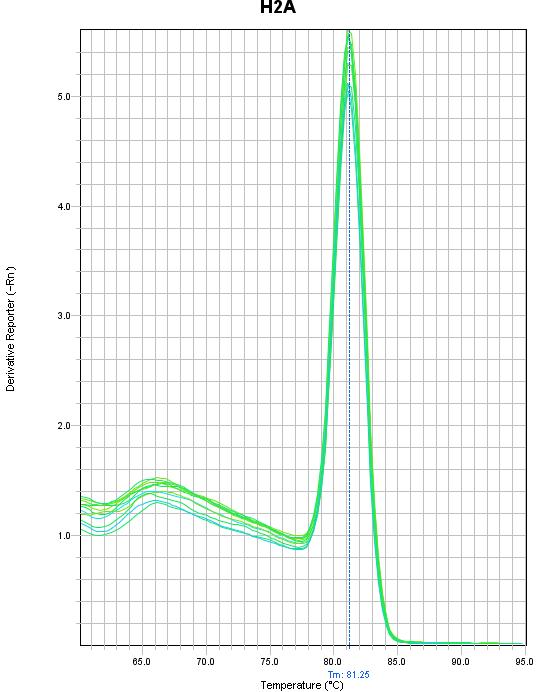

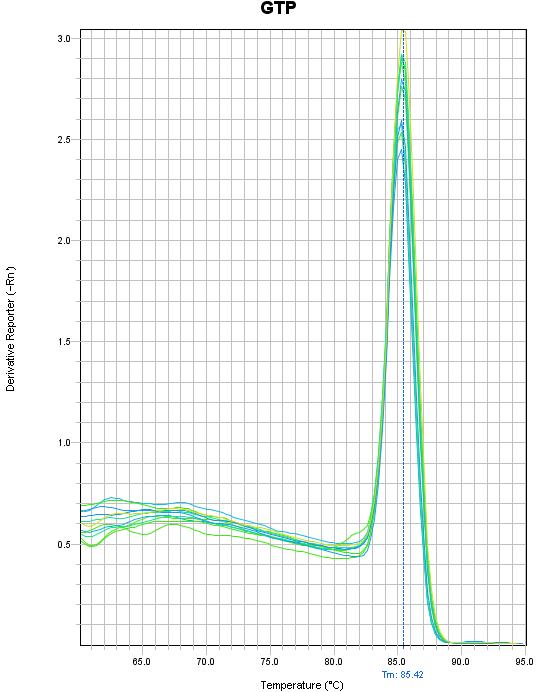

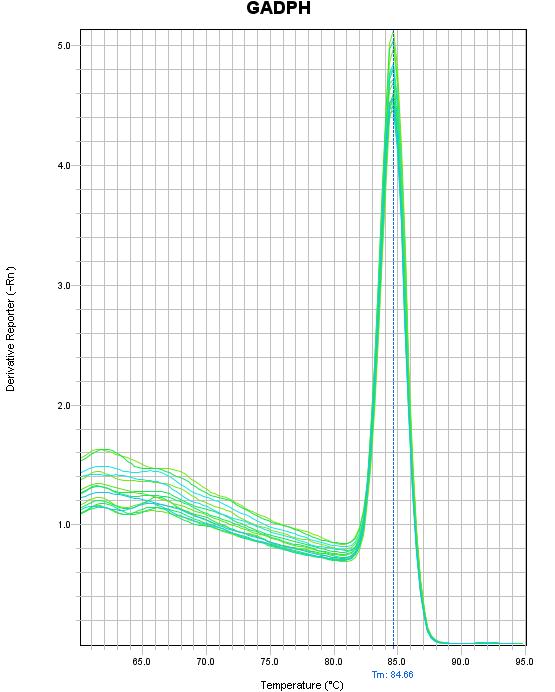

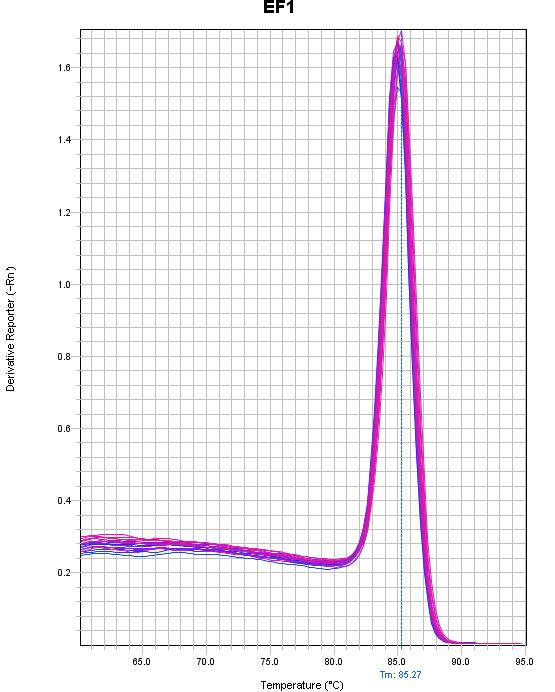

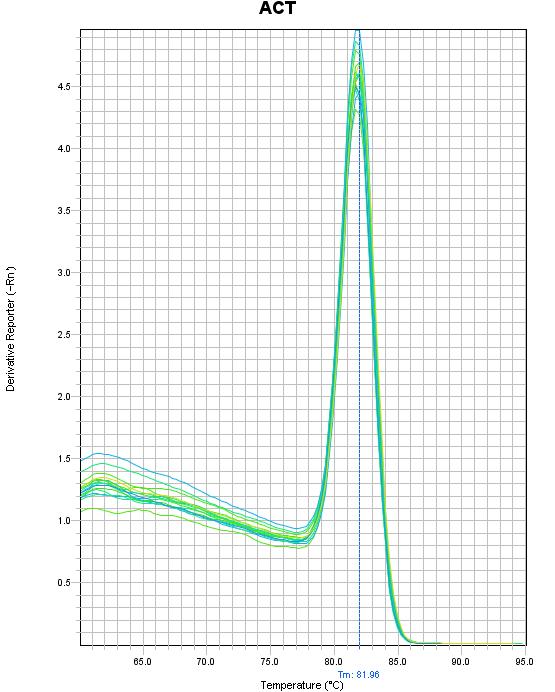

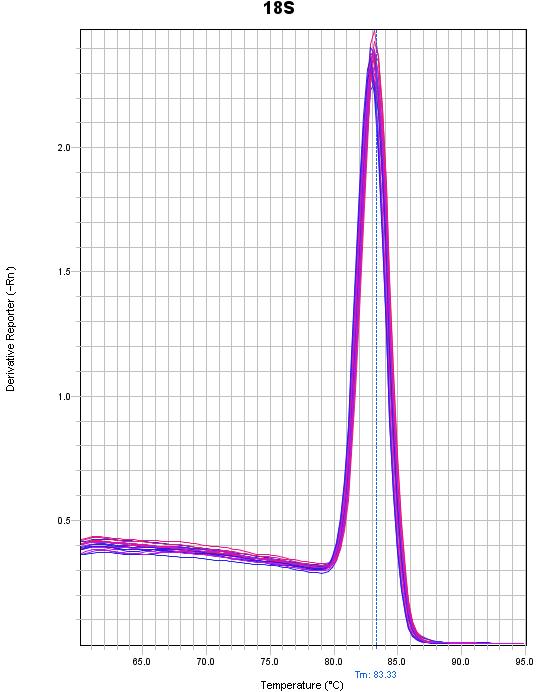

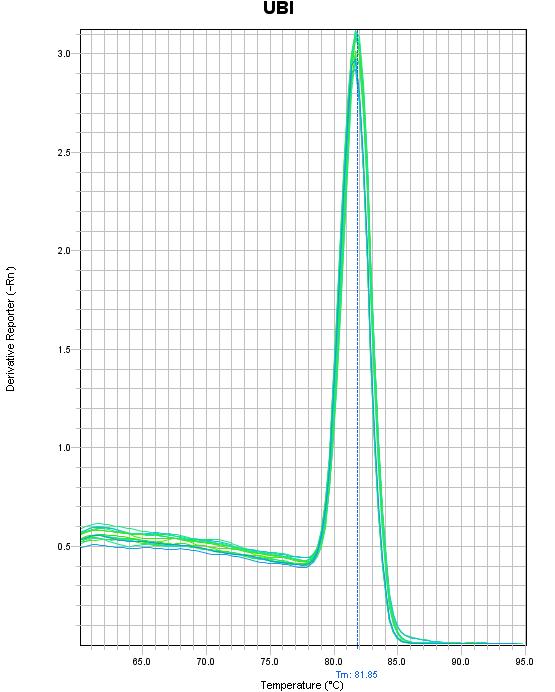

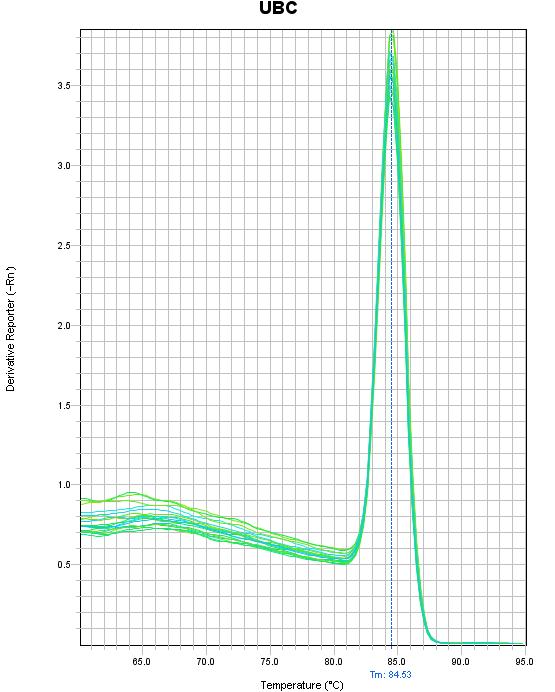


**Supplementary Figure S2：** Specificity of qRT-PCR amplicons. Dissociation curves with single peaks were generated from all amplicons and showed no dimer formation for each reference gene.
